# Large submucosal hematoma after cold snare polypectomy for colorectal adenoma

**DOI:** 10.1002/deo2.199

**Published:** 2022-12-19

**Authors:** Naomi Arakane, Kosuke Yoshida, Shoma Murata, Rika Mizuno, Koichi Furuta, Masaya Tojo, Hiroki Tamagawa, Ryoichi Miyanaga, Kazuyo Watanabe, Seiichiro Fukuhara

**Affiliations:** ^1^ Department of Gastroenterology and Hepatology National Hospital Organization Tokyo Medical Center Tokyo Japan

**Keywords:** adverse effects, cold snare polypectomy, colorectal neoplasm, endoscopic mucosal resection, mucosal hematoma

## Abstract

Cold snare polypectomy (CSP), for the treatment of colorectal polyps, has become widespread due to its low incidence of adverse events compared to that of endoscopic procedures such as endoscopic mucosal resection. However, we experienced a case of large hematoma development shortly after CSP for a colorectal adenoma despite no bleeding during the procedure. The patient underwent CSP for a 7‐mm type Isp lesion in the ascending colon. She returned the following day because of hematochezia. Computed tomography showed a 70‐mm, high‐intensity mass in the ascending colon, consistent with the large hematoma that was detected by colonoscopy. Although the patient initially had right‐sided abdominal pain, it gradually improved with conservative treatment. The hematoma decreased in size, and she was discharged 20 days after emergency admission. Although CSP can be a favorable alternative to more invasive procedures and is expected to be performed more frequently, adverse events, such as that described in this case, should be anticipated.

## INTRODUCTION

Cold snare polypectomy (CSP) has recently become a more common treatment for colorectal lesions <10 mm in size. CSP results in fewer adverse events than those resulting from endoscopic mucosal resection (EMR). Thus there have been few reports of hematoma after CSP. We experienced a case of large hematoma combined with anemia and inflammation after CSP that was not recognized during the procedure. To our knowledge, this is the first patient with this clinical course after CSP.

## CASE REPORT

**FIGURE 1:**
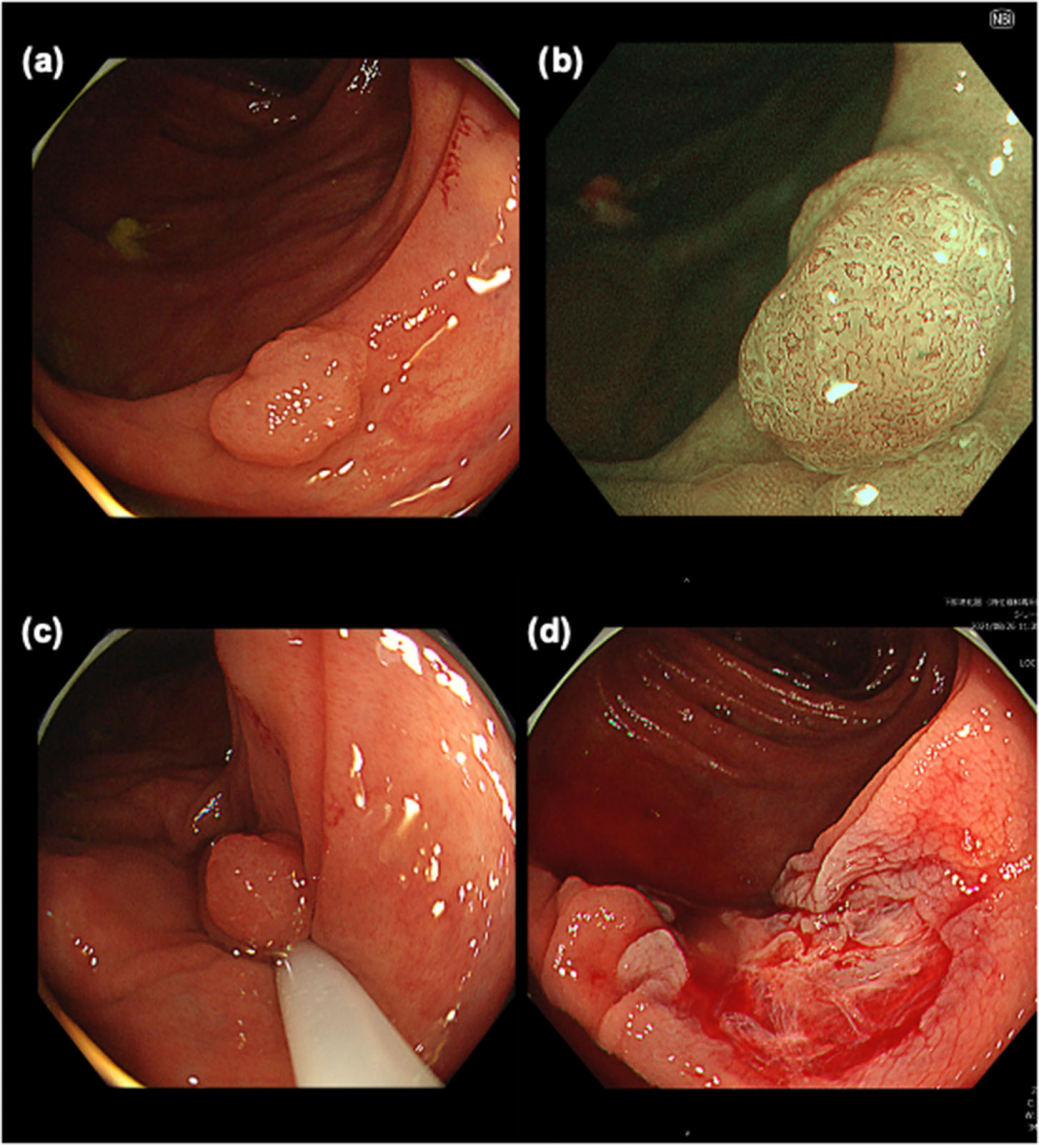
Initial endoscopic imaging findings: (a) a 7‐mm type Isp polyp detected at the ascending colon; (b) narrow band imaging‐magnified image indicates a Japan Narrow Band Imaging Expert Team (JNET) 2A pattern; (c) cold snare polypectomy has been performed with sufficient margin; and (d) no active bleeding is noted immediately after the cold snare polypectomy.

**FIGURE 2:**
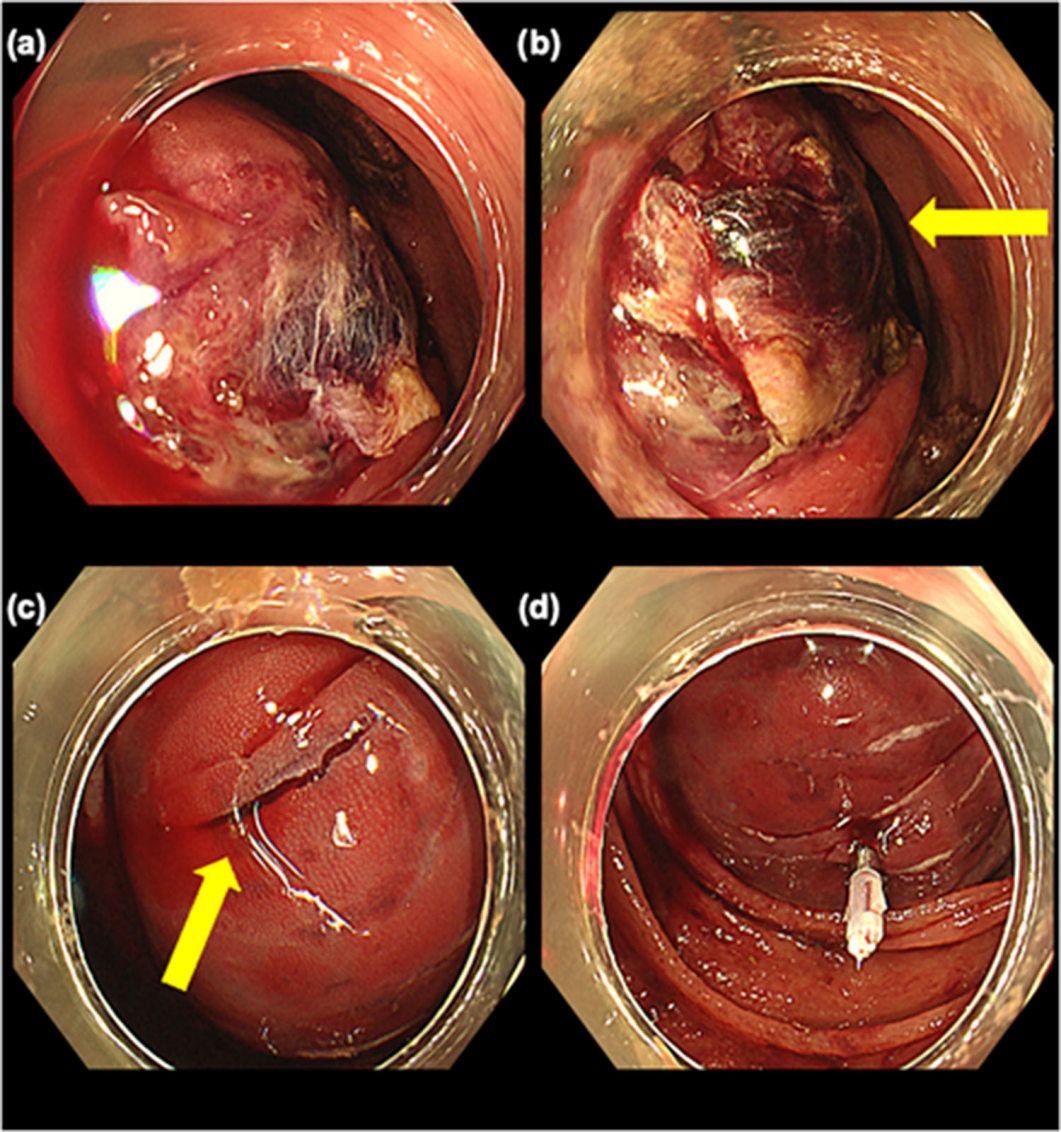
Endoscopic imaging the day after cold snare polypectomy: (a) dark and red clot attached around the lesion; (b) no visible vessels detected in the ulcer (yellow arrow indicates ulcer in hematoma); (c) partial laceration observed on the hematoma (yellow arrow indicates laceration); and (d) additional clipping performed on the laceration

**FIGURE 3:**
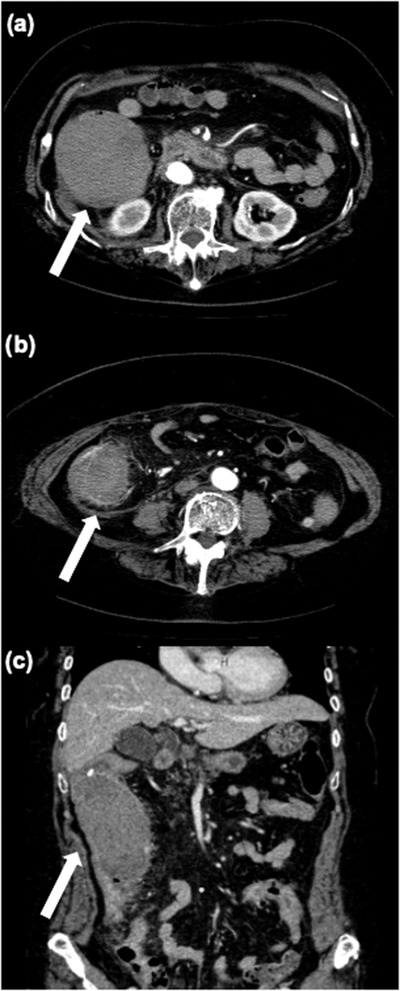
Contrast‐enhanced computed tomography performed immediately after urgent endoscopy the day after cold snare polypectomy: (a) a 70‐mm hematoma (white arrow) observed at the ascending colon; (b) a dirty fat sign visible around the ascending colon (white arrow); and (c) coronal view of dirty fat sign around the ascending colon (white arrow)

**FIGURE 4:**
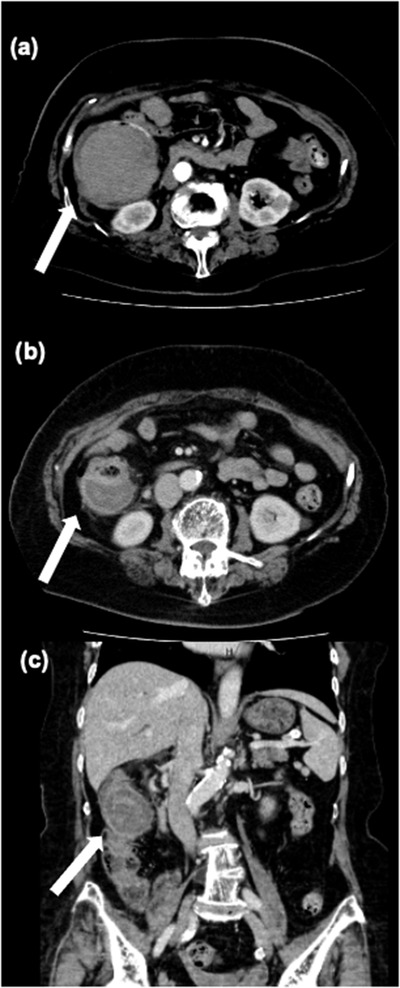
Contrast‐enhanced computed tomography 6 and 16 days after cold snare polypectomy (CSP): (a) hematoma (white arrow) 6 days after CSP, unchanged; (b) hematoma (white arrow) was decreased to about 45 mm in 16 days after CSP; and (c) coronal view of the hematoma (white arrow) 16 days after CSP

An 81‐year‐old woman was referred to our hospital because of a positive fecal occult blood test during her health checkup. She had diabetes and hypertension and was taking aspirin, famotidine, amlodipine, bisoprolol fumarate, metformin, and solifenacin succinate. Surveillance colonoscopy revealed a 7‐mm type Isp lesion in the ascending colon. Because narrow band imaging (NBI) magnification indicated The Japan NBI Expert Team 2A lesion, the lesion was diagnosed as adenoma and CSP was performed (Figure 1). Although we did not observe adverse events during the procedure, such as bleeding and perforation, the patient returned to the hospital the following day having experienced bloody stools that began the previous night. Blood examination showed marked a progression of anemia with a moderate elevation of inflammatory markers. Serum hemoglobin level (10.0 g/dl) was lower than that on presurgical examination (13.3 g/dl), whereas C‐reactive protein serum level and white blood cell count increased to 6.8 mg/dl and 13,100/μl, respectively. Considering the potential for postoperative hemorrhage, urgent colonoscopy for hemostasis was performed. Colonoscopy indicated the accumulation of giant blood clots in the ascending colon. Moreover, a large submucosal hematoma was identified in the posterior part of the lesion after CSP (Figure 2). Although there were no remarkable visible vessels in the lesion, there was partial laceration over the hematoma, wherein we assumed the bleeding was caused by the laceration area. Unfortunately, there was no photo showing the location of the ulcer and laceration. Clipping was performed to close the laceration to prevent rebleeding. After the colonoscopy, contrast‐enhanced computed tomography (CT) of the upper abdomen and pelvic region showed a 70‐mm, high‐intensity mass in the ascending colon consistent with the hematoma detected by colonoscopy. Furthermore, ascites was observed in the dorsal side of the hematoma and in the pelvis with slight inflammation around the ascending colon (Figure 3). The patient was hospitalized immediately and treated with fasting and supplemental fluids. Although she initially had right‐sided abdominal pain, it gradually improved. While serum hemoglobin level was decreased to 8.1 g/dl 2 days after CSP, hematochezia was not observed. Contrast‐enhanced CT 6 days after admission showed that intramucosal hematoma was not increased without new ascites (Figure 4). Therefore, the patient resumed food intake. CT 16 days after admission revealed further reduction of the hematoma (Figure 4). Although the elevation of serum C‐reactive protein level was persistent due to concomitant occurrence of cholangitis, it decreased to 0.98 mg/dl on day 10 after endoscopic treatment. Meanwhile, white blood cell level decreased to 8100/μl 3 days after CSP. As there was no recurrence of abdominal pain after the patient resumed eating, and anemia improved after admission, the patient was discharged 20 days after admission. Serum hemoglobin levels improved to 10.6 g/dl after discharge.

## DISCUSSION

We report a rare case of large hematoma development shortly after CSP for a colorectal adenoma despite no bleeding during the procedure. CSP can be used to treat polyps <10 mm without energization and is known to have less delayed submucosal vascular injury due to thermal coagulation action on the mucosal muscle plate, thereby exerting lower risk of postprocedural hemorrhage and perforation. A meta‐analysis, including 8 studies and 1665 patients who underwent CSP or hot snare polypectomy (HSP), noted that delayed bleeding occurred only in patients who underwent HSP.^1^ Other meta‐analyses showed that delayed bleeding was more common in patients undergoing HSP than in those undergoing CSP, but the difference was not significant.^2^

In the present case, the patient had hematochezia on the night following CSP operation. However, there was no bleeding during the endoscopic observation immediately after CSP. Negishi *et al.* noted a massive hematoma after CSP but did not mention any procedural factors that may have contributed to hematoma formation.^3^ Although the report presents a similar case to the present study, endoscopic treatment techniques were utilized; thus, the detailed course of the treatment was unclear.

The patient in this case was taking aspirin, an antithrombotic agent. A previous retrospective study showed that there was no significant difference in the incidence of delayed bleeding among patients who did and did not receive antithrombotic drugs.^4^ However, another report revealed that posterior hemorrhage was more common in patients receiving oral antithrombotic medication.^5^ Although one study examined only direct oral anticoagulants, the findings suggest that the risk of posterior bleeding can be reduced by discontinuing medication on the day of the procedure.^6^ Guidelines from the Japan Gastroenterological Endoscopy Society allow endoscopic procedures, especially CSP to be performed if anticoagulants are rested for 24 h.^7^ However, aspirin can be continued. Although CSP was performed while patients were still taking aspirin in accordance with the guidelines, caution should be exercised regarding bleeding associated with endoscopic procedures. We must also consider the possibility of bleeding, even in low‐risk procedures such as CSP.

Although CSP is thought to cause little damage to the submucosal layer, a study of resected specimens indicated that approximately one quarter of samples included the submucosal layer.^8^ Moreover, a previous report using the pig colon revealed that CSP with pull technique can resect mucosal layer, muscularis mucosa, and upper submucosal layer.^9^ It is possible that the hematoma formed in the subserosal layer beyond the submucosa as shown on the CT scan after CSP. Although the submucosa or deeper layers were not included in the resection specimen in this case, and it is difficult to assess which layers were resected, the possibility cannot be ruled out that the CSP procedure may have disrupted the deeper vessels.

Damage to the submucosal or deeper layers may have contributed to hematoma formation. Moreover, the patient had moderate inflammation around the ascending colon with an accumulation of ascites, suggesting the influence of a deep layer of the colonic wall. Therefore, CSP with pull technique might cause the formation of a large hematoma through damage to the submucosa and deeper layers, such as the muscle and subserosal layers. It was supposed that inflammation deeper than the submucosa resulted in localized enteritis, with elevated C‐reactive protein and peritonitis with ascites.

There are several limitations associated with the present study. We experienced this rare event in only one case. Although we mentioned the procedural action of the snare, it is difficult to determine how the large hematoma was induced, including the influence of anti‐platelet agent aspirin. Further investigation into the etiology of hemorrhage and hematoma after CSP will be possible with more cases.

In conclusion, we experienced a case of large mucosal hematoma after CSP. Although CSP has a lower risk of adverse events than other procedures, such as EMR, it is important to be aware of the potential adverse events, especially for patients receiving antithrombotic agents.

## CONFLICTS OF INTEREST

None.
